# Case Report of Cutaneous Squamous Cell Carcinoma at the Wrist Joint and the Public Health Crisis of Arsenicosis

**DOI:** 10.5696/2156-9614-11.29.210314

**Published:** 2021-03-02

**Authors:** Sonal Sachan, Sucheta Pathania, Abbas Ali Mahdi, Swastika Suvirya, Atin Singhai

**Affiliations:** 1 Department of Dermatology, Venereology and Leprosy; King George's Medical University, Lucknow, Uttar Pradesh, India; 2 Department of Dermatology, Venereology and Leprosy; Zonal Hospital, Dharamshala, Himanchal Pradesh, India; 3 Department of Biochemistry; King George's Medical University, Lucknow, Uttar Pradesh, India; 4 Department of Pathology; King George's Medical University, Lucknow, Uttar Pradesh, India

**Keywords:** arsenicosis, palmo-plantar hyperkeratosis, squamous cell carcinoma

## Abstract

**Context.:**

Arsenicosis is caused by long term (6 months plus) ingestion of arsenic above a safe dose, characterized by skin lesions and possible involvement of internal organs. Arsenicosis is common in India and Bangladesh where naturally occurring high concentrations of arsenic in the earth's crust contaminate ground water, causing adverse health effects.

**Case Presentation.:**

We report a case of a 55-year-old Indian male, resident of a known arsenic endemic region of Uttar Pradesh who suffered from characteristic pulmonary and cutaneous features of chronic arsenic toxicity which included radiological findings of interstitial lung disease, hyperkeratotic lesions over the palms and soles, rain drop like pigmentation over the trunk, and carcinomatous changes at the wrist joint. The patient was started on chelating agents (d-penicillamine) and oral retinoids (isotretinoin) followed by the surgical excision of the carcinoma.

**Discussion.:**

Environmental contamination with arsenic is a well-known health hazard in South Asian countries. The main source is consumption of contaminated ground water for domestic purposes. Cutaneous lesions, internal organ involvement including interstitial lung disease and carcinomas as observed in our patient have been reported in the literature. Various mechanisms like epigenetic changes and arsenic-induced immune suppression have been proposed for the development of cutaneous carcinomas with prolonged exposure to arsenic.

**Relevance to Clinical Practice.:**

Among the various causes of palmo-plantar hyperkeratosis, arsenicosis should be kept in mind when presenting in combination with pigmentary changes and carcinomatous growth from an arsenic-endemic region.

**Conclusions.:**

People residing in arsenic-endemic regions should be made aware of arsenic-related health hazards. Rainwater harvesting and good nutrition are the simplest measures which could be adopted by the exposed population in affected areas. Several methods have also been employed by governmental and non-government organizations to separate arsenic from contaminated water to combat arsenic-related diseases and carcinomas.

**Competing Interests.:**

The authors declare no competing financial interests.

## Context

The main source of arsenic exposure for humans is contaminated ground water, which is used for domestic purposes across various regions of India. Arsenic contamination occurs through natural mineral deposits as well as anthropogenic sources like mining activities, electronic manufacturing processes, smelting of metals and pesticides used in farming.^1,2,3^ Arsenic contamination of ground water is a major public health concern. Acute arsenic poisoning is associated with nausea, vomiting, abdominal pain, and severe diarrhea, while chronic arsenic toxicity is characterized by multi-system involvement and cancers of various organs like the lungs, liver, bladder, and skin.^3^

In India, chronic arsenic toxicity was first reported in Chandigarh in 1976 and later from the state of West Bengal in 1984.^1,3^ There have been several reports of groundwater arsenic contamination in states situated on the Ganga-Brahmaputra and the Padma-Meghna fluvial plains.^2^ These states are Assam, Bihar, Chhattisgarh, Jharkhand, Manipur, Uttar Pradesh, and West Bengal, where the arsenic concentration in groundwater was above 10 μg/L (greater than the World Health Organization's (WHO) recommended level.^3,4^ Moreover, indiscriminate pumping of ground water in the northern states of India has led to a large increase in arsenic concentrations beyond safe levels. There are also reports documenting elevated levels of arsenic in tube well water samples in the area. People residing in these arsenic-affected areas manifest various types of cutaneous diseases including ulcers, pigmentation, hardening of palmar skin and various cancerous growths in the body.^5^ Arsenicosis is a preventable condition if diagnosed at an early stage. By reporting this case we are highlighting a serious public health crisis caused by environmental arsenic contamination in some known arsenic-endemic areas in India. While cases of acute arsenic poisoning have been well documented, to the best of our knowledge, this is the first case report of a patient with elevated blood arsenic levels and symptoms of chronic arsenic toxicity without the symptoms of acute toxicity.

## Case Presentation

**Figure 1:**
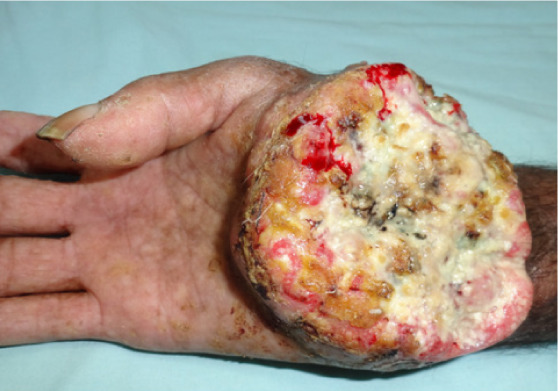
A well defined ulcero-proliferative fungating growth of size 8 cm × 7 cm, with elevated margins and covered with yellowish, foul smelling discharge on the volar aspect of the right wrist

**Figure 2:**
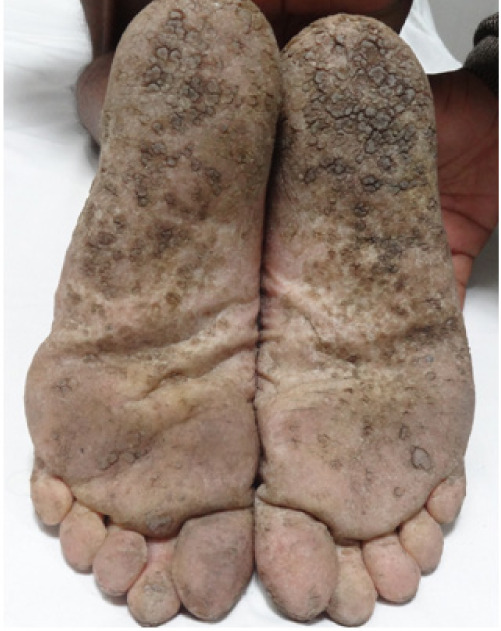
Multiple variable sized thick, rough keratotic papules on the ventral aspect of the feet

A 55-year-old male, farmer by occupation, presented to us with the chief complaints of rough raised lesions over the palms and soles for the last 3–4 years, hyper and hypo-pigmented macules over the trunk, upper and lower limbs for last 2–3 years and a painful ulcer over the right wrist for the last 2 years. He also had a history of cough, shortness of breath, on and off paresthesia on the upper right limb, hypertension, and weight loss (undocumented). To treat these symptoms, the patient took multiple over-the-counter medications for pain (undocumented), but his condition continued deteriorating. Various social factors were responsible for the patient's hesitancy to seek treatment for his symptoms, including illiteracy, lack of understanding of the seriousness of his symptoms, financial difficulties, and lack of access to good medical facilities in his area of residence. On further inquiry, the patient revealed a history of consumption of ground water (hand-pumped) for domestic purposes. The patient was a farmer by occupation and did not report any history of occupational exposure to arsenic contaminated aerosols/dust related to mining or industrial activities, and no such mines or industries were located near his residence. There was no history of similar complaints among others in the same locality, past intake of indigenous medicine, headache, weakness, easy fatiguability, insomnia, memory loss, tremors, vision loss, anorexia, nausea, vomiting, diarrhea, abdominal pain or mucosa pigmentation. Written informed consent for physical examination, clinical pictures and biopsy was obtained from the patient. On clinical examination there was a single well-defined, ulcero-proliferative growth of size 8 × 7 cm, with elevated margins, covered with yellowish, foul smelling discharge, tender and fixed to the underlying structures on the volar aspect of the right wrist (*Figure 1*). Multiple hyperkeratotic papular lesions of size 4–5 mm were present on both palms and soles (*Figure 2*). Hyper and hypo-pigmented macules were present on the trunk, upper and lower limbs. A single, 3 × 2 cm, mobile, hard, non-tender lymph node was present in the right axilla (central group). No organomegaly was noted. Keeping in mind the differentials like arsenicosis, plantar and palmar warts and epidermodysplasia verruciformis, several investigations were performed.

**Figure 3:**
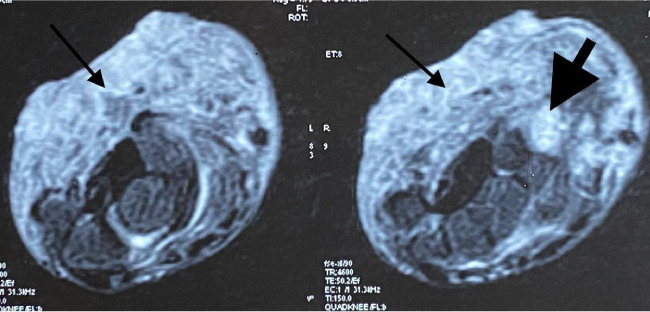
Magnetic resonance imaging images of the right wrist (short tau inversion recovery (STIR) axial) showing an ill-defined soft tissue lesion involving the flexor aspect of the distal third of the forearm and wrist (thin black arrow). Lesion involving the pisiform bone (thick black arrow).

**Figure 4:**
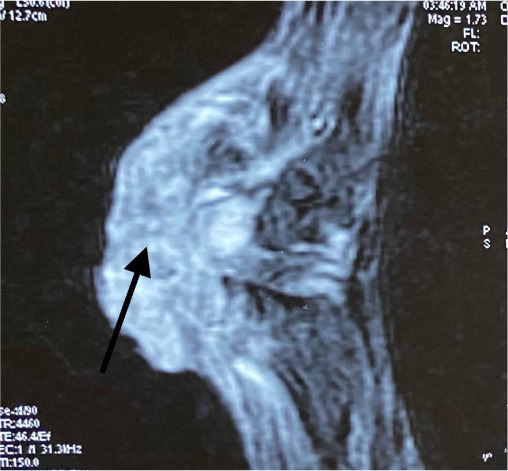
MRI images of the right wrist- (STIR sagittal) showing an ill-defined soft tissue lesion involving the flexor aspect of the distal third of the forearm and wrist (thin black arrow). Lesion involving the pisiform bone (thick black arrow).

**Figure 5:**
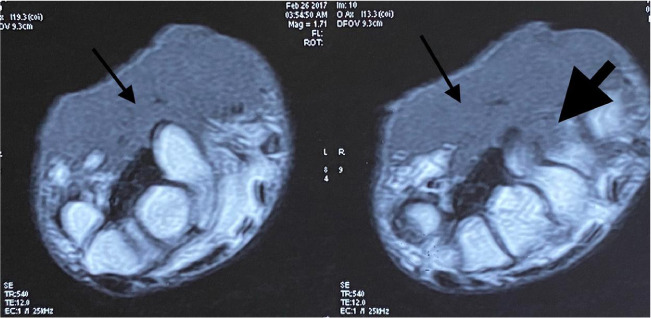
MRI images of the right wrist- (T1WI axial) showing an ill-defined soft tissue lesion involving the flexor aspect of the distal third of the forearm and wrist (thin black arrow). Lesion involving the pisiform bone (thick black arrow).

**Figure 6:**
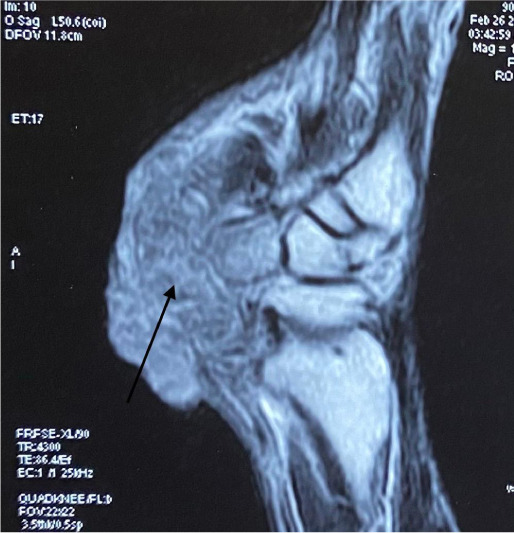
MRI images of the right wrist (T1WI sagittal) showing an ill-defined soft tissue lesion involving the flexor aspect of the distal third of the forearm and wrist (thin black arrow). Lesion involving the pisiform bone (thick black arrow).

**Figure 7:**
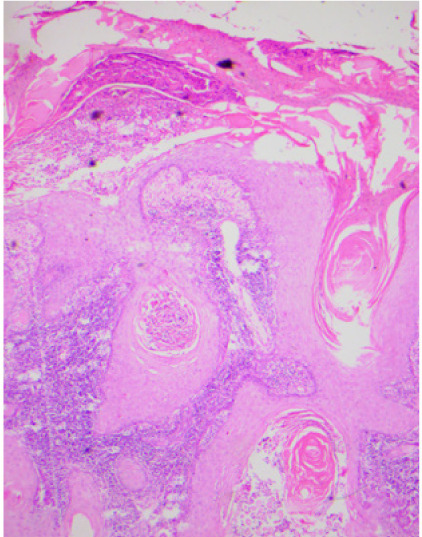
Hematoxylin and eosin-stained biopsy section showing acanthotic, dyskeratotic, squamous epithelial lining with orthokeratotic hyperkeratosis as well as large cell keratinizing squamous cell carcinoma (40x).

His hemogram, liver and renal function tests, blood sugar and serum lipid profile were within normal limits. Metal analysis was carried out by inductively coupled plasma-optical emission spectrometry (ICP-OES) after microwave digestion of samples as described method by Ansari *et al.*, 2015 with a slight modification.^6^ Briefly, blood, hair and nail samples were acid digested in a microwave reaction system (Multiwave 3000, Anton Paar, Perkin Elmer, USA) and the resultant clear solution was analyzed by ICP-OES using a low flow system (Perkin Elmer, Optima, 8000, USA).^6^ On metal analysis, arsenic levels in whole blood were found to be 29 mcg/dl (normal range, 0.03–0.2 mcg/dl), while levels of other metals like lead, mercury and cadmium were within normal limits.^7^ Hair and nails did not reveal elevated metal levels. Right ulnar neuropathy was found on the nerve conduction study. Magnetic resonance imaging (MRI) of the right wrist showed a soft tissue lesion involving the flexor aspect of the distal third of the forearm and right wrist (*Figure 3, 4, 5, 6*). High resolution computed tomography (HRCT) of the chest showed hyper-inflated bilateral lung with patchy fibrotic changes and focal pleural thickening in bilateral upper lobes with bronchiectatic changes in the right middle lobe with tiny nodules in the bilateral lung fields. His electrocardiogram and electroencephalogram were normal. Histopathological examination from the papular lesions over the palms revealed hyperkeratotic, hypertrophic stratified squamous epithelium with parakeratosis, and the underlying squamous epithelium zone showed fibro-collagenous tissue with mild inflammatory lymphomononuclear cells. Biopsy from the edge of the ulcer at the right wrist showed malignant epithelial neoplasms in clusters and sheets. Individual cells were pleomorphic and medium-sized with high nucleocytoplasmic ratio, enlarged vesicular nuclei, occasional prominent nucleoli with scanty cytoplasm, and a few keratin pearls were also seen (*Figure 7*). On the basis of the above findings, diagnosis of arsenicosis with squamous cell carcinoma on the right wrist joint was made. Possibilities of warts and epidermodysplasia verruciformis were ruled out. The patient was started on oral d-penicillamine 250 mg, folic acid 5 mg and isotretinoin 20 mg with topical 10% urea cream and 6% salicylic acid cream to be applied on the keratotic lesions.

**Figure 8:**
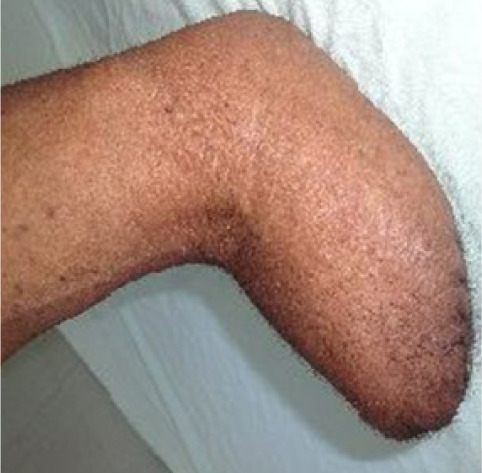
Healthy stump (after below elbow amputation)

Right forearm amputation was advised by the surgeon for the carcinomatous ulcer at the wrist joint of the same side. At first this treatment was unacceptable to the patient as his dominant hand had to be amputated. His difficulty comprehending the seriousness of his diagnosis owing to illiteracy and compounded by financial constraints led to delay of his surgical amputation by six weeks after starting his oral medications. Almost six weeks later, when the patient was both mentally and financially prepared, right below elbow amputation along with right axillary lymph node dissection was performed after obtaining consent. Histopathological examination of the growth after right below elbow amputation showed well differentiated large cell keratinizing invasive squamous cell carcinoma with clear margins. The stump was found to be healthy at one month follow-up (*Figure 8*).

D-penicillamine was given for a total duration of two weeks. The patient's poor financial background did not allow us to monitor his blood and urine arsenic levels during therapy with d-penicillamine. Not much improvement in palmo-plantar hyperkeratosis was evident after three months of isotretinoin therapy. Hence, owing to no further improvement and considering the high cost of isotretinoin, it was stopped at the end of the third month. Counseling was given regarding use of alternative sources of water. The patient was continued on topical therapy with no fresh complaints on subsequent follow-up visits.

## Discussion

The patient in this case report came from a region of endemic arsenic toxicity in the Ballia district situated in Uttar Pradesh, a densely populated northern state of India.^8^ In the Ballia district, arsenic is reported to be found in Holocene sediments of the active river system.^9^ A preliminary clinical examination in 11 affected villages (ten from Ballia and one from the Gazipur district of Uttar Pradesh state) revealed typical arsenical skin lesions ranging from melanosis, keratosis to Bowen's disease.^10^

Arsenic can enter the food chain through air, water, and soil. It is principally consumed as arsenite (As^+3^) and arsenate (As^+5^). Reduction and oxidative methylation occur following absorption in the body and is often excreted through urine. The mechanism of toxicity and organ involvement depends on the valence state of arsenic. Arsenite (As^+3^) is known to bind with keratins and get deposited in skin, hair, nails, and the gastro-intestinal system, while arsenate (As^+5^) is deposited in bones.^11^ In the present case, the patient reported consuming ground water for the last 10 to 15 years.

Skin lesions are the earliest manifestations in affected patients as was the case with the patient reporting to us. Cutaneous manifestations are the most prominent characteristic finding used in identifying arsenicosis patients.^3,12^ Keratosis on the soles is suggested to be the most sensitive marker for detection of arsenic toxicity at an early stage. Keratotic lesions are classified on the basis of size into mild (<2 mm), moderate (2–5 mm) and severe (>5 mm) forms. Confluent keratotic plaques can also be present.^1^ We observed that hyperkeratotic lesions over the palms and soles were the first manifestations in our patient. A retrospective review of the dermatologic manifestations of chronic arsenic poisoning found that arsenic keratosis and dyspigmentation are the most common findings.^13^ Moreover, melanosis is another commonly encountered feature. It can present in the form of rain drop pigmentation (freckle-like) or diffuse pigmentation.^13^ In our patient, rain drop pigmentation was observed on the trunk. Although not found in this patient, mucosal pigmentation can also be present.^13^

Pulmonary involvement is known to occur with arsenic-contaminated ground water used for drinking purposes, which is further supported by the literature describing increased levels of arsenic in bronchio-alveolar lavage fluid collected from patients who developed diffuse interstitial lung disease due to consumption of arsenic-contaminated drinking water for prolonged duration.^14–20^ Therefore, consumption of arsenic-contaminated ground water by our patient was considered the possible cause for lung involvement after ruling out exposure to arsenic via inhalation due to mining or industrial activities.^14–20^ According to the literature, patients with palmo-plantar hyperkeratosis and/or cutaneous cancers were more susceptible to developing respiratory disease due to chronic arsenic exposure, and similar findings were noticed in our patient.^21^ The common symptoms are cough, sputum as well as dyspnea, and our patient presented with cough and dyspnea.^21^ The HRCT findings of respiratory involvement may take the form of diffuse interstitial lung disease, bronchiectasis, pulmonary nodules, or bulla-emphysema.^21^ Of these, bilateral pulmonary patchy fibrosis and bronchiectatic changes were noticed on HRCT of the chest in our patient. Although peripheral neuropathy is also reported in arsenicosis, in our case right ulnar neuropathy was found which was most likely due to compression caused by the carcinomatous changes on the right wrist joint.^3^

Other major health issues resulting from chronic arsenic exposure are anxiety, irritability, sleep disorders, peripheral vascular diseases causing ischemic limbs and gangrene. Although there are limited data from India, cardiovascular disorders can also occur. Anemia and diabetes mellitus have also been reported. Adverse pregnancy outcomes like spontaneous abortion, preterm birth and stillbirth have been reported from the affected regions. Data show that infants and children are more susceptible to chronic arsenic poisoning which affects their physical growth as well as mental development.^3^

Arsenic is a known carcinogen. Numerous studies report associations between arsenicosis and skin cancer.^22–25^ Squamous cell carcinoma and multiple basal cell carcinoma are typical arsenic-induced skin cancers, while Bowen's disease indicates impending skin cancer.^3^ Our patient also presented with squamous cell carcinoma at the wrist joint along with other cutaneous findings. The major characteristic of arsenic-induced cutaneous squamous cell carcinoma is that they appear at sun-protected areas of the body as observed in our patient (volar aspect of the right wrist joint).^26^ Several mechanisms have been proposed for arsenic-induced carcinogenesis. The first involves changing the epigenome, thus altering the chromatin structure and dynamics. These epigenetic changes act at the level of transcription initiation as well as at gene splicing level thus altering the gene regulatory factors. Chromosomal instability and epigenetic modifications are the proposed factors leading to carcinogenicity following arsenic exposure. Deoxyribonucleic acid (DNA) methylation, post-transcriptional modifications (PTMs) of histone proteins like methylation, acetylation, glycosylation, phosphorylation, etc. are some of the epigenetic mechanisms which finally lead to carcinogenicity. Another epigenetic mechanism of gene regulation affecting growth, development and response to stress are microribonucleic acids (miRNAs). Arsenic exposure leads to alteration in miRNA gene expression. It affects the kinetics of polymerase elongation and recruitment of splicing regulatory factors, leading to carcinogenicity.^27,28^

There are reports that arsenic exposure also leads to immune suppression by acting on T and B cells as well as on macrophages. There is reduced expression of major histocompatibility complex (MHC) class II molecules, CD69, interleukin-1 beta (IL-1β) and tumor necrosis factor-alpha (TNF-α); decreased lymphocyte proliferation, migration and IL-2 secretion; impaired macrophage adhesion, phagocytosis and increased apoptosis of PBMC (peripheral blood mononuclear cells), and decreased stimulated reactive oxygen species (ROS) production by PBMC. By affecting both cellular and humoral immunity, arsenic exposure leads to an immune-compromised state and finally malignancy along with other effects.^29^

Chronic arsenic ingestion leads to its accumulation in several organs including the lungs and skin, for which chelation therapy is recommended based on a few individual cases and clinical expert opinion in order to reduce the arsenic burden from these organs and decrease the level of arsenic in blood by excreting it out through urine.^1,5,20,30–33^ As there are no proper treatment protocols for prescribing d-penicillamine in acute as well as chronic arsenic poisoning, our patient, who was found to have raised arsenic blood levels, was given d-penicillamine for two weeks without monitoring blood and urine arsenic levels during and after chelating therapy because of the social factors outlined earlier.^1,20,30–33^ Although there are mixed responses to d-penicillamine when used for chronic arsenic poisoning, it is preferred over other chelators like dimercaprol because of its ease of administration and better availability.^1,30,31^ Several studies have shown beneficial effects of oral retinoids in treatment of chronic arsenic poisoning induced palmo-plantar hyperkeratosis, considering their anti-keratinizing role in several keratinization disorders.^1,20,34,35^ Another advantage of using oral retinoids is that they can be used as a chemopreventive medication for arsenic-related cancers, by altering gene expression.^1,34,35^ Among oral retinoids, acitretin has generally been used for arsenic keratosis but due to easy availability, lower cost and better side effects profile, we preferred isotretinoin.^1,34,35^ Hence, along with d-penicillamine we used oral isotretinoin in our patient. Both of these drugs have shown good outcomes in a few individual cases, but in the present case, it did not have much clinical response and considering the patient's financial limitations, both medications were stopped after their respective duration of therapy. We counseled the patient on nutrition improvements and use of alternative water sources in order to avoid further damage to his health. We invited nearby residents to visit us for free health check-ups to check for further arsenic-related manifestations. Simultaneously, we worked to raise awareness regarding the health risks of ground water contaminated with heavy metals which was prevalent in their area. Finally, a neighbor in a better financial position agreed to provide reverse osmosis treated water free of cost to our patient for drinking purposes.^36^

## Conclusions

Cases of chronic arsenic poisoning continue to rise in India despite multiple programs by governmental and non-government organizations to mitigate the adverse health effects of arsenic.^37^ Newer arsenic remediation technologies have been either approved or under consideration through these programs. Arsenic toxicity can be prevented by raising awareness of this condition. Education programs are needed to address unsafe water sources and methods for removing arsenic from contaminated water. Filtration techniques (reverse osmosis) and rainwater harvesting methods should be employed to avoid use of contaminated ground water.^36,37^ Good nutrition has also been reported to be effective in combating the adverse effects of chronic arsenic exposure.^3^

After recovering from surgery and adopting an alternative source of water for drinking purposes, our patient's health improved. After recovery, the patient and his wife returned to their employment in the fields. The patient improved his diet after receiving nutrition counseling. Through all of these efforts, the hazardous health-related effects of arsenic toxicity resulting in interstitial lung disease and carcinoma as observed in our patient can be minimized in an exposed population.
